# Vascular malformations of the head and neck and a molecularly guided precision therapy framework

**DOI:** 10.3389/fneur.2026.1796746

**Published:** 2026-03-18

**Authors:** Hui-Qi Qu, Dong Li, Hakon Hakonarson

**Affiliations:** 1 The Center for Applied Genomics, Children’s Hospital of Philadelphia, Philadelphia, PA, United States; 2 Department of Pediatrics, The Perelman School of Medicine, University of Pennsylvania, Philadelphia, PA, United States; 3 Division of Human Genetics, Children’s Hospital of Philadelphia, Philadelphia, PA, United States; 4 Division of Pulmonary Medicine, Children’s Hospital of Philadelphia, Philadelphia, PA, United States; 5 Faculty of Medicine, University of Iceland, Reykjavik, Iceland

**Keywords:** etiological therapy, genomics, head and neck, PI3K-AKT–mTOR, precision medicine, RAS-MAPK, targeted therapy, vascular malformations

## Abstract

Vascular malformations of the head and neck carry high morbidity because of their proximity to the airway, cranial nerves, sensory organs, and cerebral vasculature. Although the ISSVA classification of vascular anomalies has standardized diagnostic terminology, therapeutic decisions for malformations have remained largely phenotype-driven and procedure-centered. Recent advances in genomics reveal that many vascular malformations are genetically driven disorders of endothelial signaling, enabling a transition toward etiological, mechanism-based therapy. This review presents a molecularly guided precision framework for the management of head and neck vascular malformations, focusing specifically on etiological medications that modify underlying disease biology. We synthesize evidence supporting three principal therapeutic classes: mTOR inhibition for PI3K-AKT–mTOR-driven disease, direct PI3Kα inhibition for *PIK3CA*-mediated pathology, and MEK inhibition for RAS/MAPK-driven lesions. We summarize translational and clinical evidence and provide practical guidance on indications, expected responses, toxicity monitoring, and sequencing for head and neck lesions. By reframing treatment selection around molecular drivers rather than descriptive phenotype, this review introduces a clinically actionable paradigm that advances care for vascular malformations from lesion control toward durable, mechanism-based disease modification.

## 1 Foundations of etiological therapy in vascular malformations

The classification system developed by the International Society for the Study of Vascular Anomalies (ISSVA) provides an essential and internationally consistent framework for the diagnosis of vascular malformations by organizing these disorders according to hemodynamic behavior and combinations of affected vessel types, including venous, lymphatic, arteriovenous, and complex combined malformations (1, 2). The resulting standardized nomenclature has facilitated interdisciplinary communication by enabling radiologic, pathologic, and clinical descriptions of lesions to converge on shared diagnostic terms, which in turn has strengthened clinicopathologic correlation and multidisciplinary decision-making. However, as molecular and genetic insights into vascular malformations have expanded, a central limitation of this phenotype-based system has become clear: descriptive labels do not consistently correspond to the molecular mechanisms that govern therapeutic response. Recent ISSVA updates emphasize that molecular findings increasingly inform management, and classification is evolving alongside therapeutic implications (2).

ISSVA categories are constructed from clinical appearance, imaging behavior, histopathology, and anatomic distribution (2). These elements remain indispensable for diagnosis and procedural planning, yet they offer limited guidance when the goal is to modify disease biology. Lesions with similar clinical features, or even the same ISSVA label, may be driven by distinct molecular mechanisms, most prominently dysregulation of the PI3K-AKT–mTOR or RAS–RAF–MEK–ERK pathways, with fundamentally different therapeutic sensitivities. Further complicating phenotype-based inference is the high prevalence of somatic mosaicism in vascular malformations; actionable variants are frequently present at low allele fractions and may only be detectable with appropriate tissue selection or sufficiently sensitive assays. Procedural options (sclerotherapy, embolization, surgery, and laser) may be incomplete or carry substantial risk, and recurrence is common. These are the scenarios where disease-modifying drug therapy matters most.

The need for a mechanism-guided therapeutic approach is particularly pronounced in vascular malformations of the head and neck, where biologic drivers such as PI3K pathway and RAS/MAPK pathway activation frequently underlie lesions that produce disproportionate functional consequences despite modest anatomic extent (3). Malformations involving the face, oropharynx, or skull base may impair swallowing, hearing, or vision through mass effect or direct involvement of critical neurovascular and lymphatic pathways, and periorbital arteriovenous malformations have been associated with severe vision loss (3). Because these lesions often progress in response to their molecular signaling milieu rather than purely structural factors, histologically benign disease can behave aggressively when located near the airway or sensory organs. Procedural interventions in this region also carry elevated risk owing to proximity to major vessels, cranial nerves, and cosmetically sensitive structures, and they frequently fail to address the underlying signaling abnormality. These features create a strong rationale for etiological medical therapy aimed at suppressing pathway-driven activity, stabilizing progression, and improving the safety and effectiveness of necessary procedural interventions.

Vascular malformation care has begun to shift from a diagnosis-first, procedure-first paradigm toward pathway-based treatment selection in which identification of the dominant signaling axis, most commonly PI3K pathway or RAS/MAPK pathway, guides targeted therapeutic selection. Large-scale genomic investigations have accelerated this transition by demonstrating at clinically meaningful scale that actionable variants can be detected across diverse vascular malformation phenotypes and that these findings can directly alter management. Among these, the study by Li et al. (4) provides one of the most comprehensive demonstrations of an integrated genomics-to-treatment workflow, linking molecular diagnosis with targeted therapeutic decision-making across a broad spectrum of vascular malformations. This work represents a pivotal demonstration of feasibility rather than the sole foundation of the emerging approach. In parallel, therapy-specific clinical studies further substantiate the principle that pathway inhibition can be disease-modifying, including prospective evidence supporting sirolimus in complicated vascular malformations (5) and translational and clinical evidence supporting alpelisib in lymphatic malformations (6). These data support a central premise of this review: etiological medications are most rational and most effective when guided by molecular mechanism, whether established through direct variant identification, pathway inference, or both.

This review focuses specifically on potential targeted medications for vascular malformations of the head and neck, therapies intended to modify underlying disease biology rather than solely palliate symptoms. We emphasize three major therapeutic classes that currently anchor etiological treatment in clinical practice: mTOR inhibition (e.g., sirolimus and everolimus), PI3K inhibition (e.g., alpelisib for *PIK3CA*-driven disease), and MEK inhibition (e.g., trametinib for RAS/MAPK-driven disease) (Table 1). These therapies are examined within a molecularly guided approach that highlights the practical determinants of real-world success, including patient selection, specimen strategy in the setting of mosaicism, pathway assignment, response assessment, treatment sequencing and switching logic, and long-term safety monitoring. Procedural modalities are discussed only in contexts where they intersect with pharmacologic strategy, including neoadjuvant or adjunctive use of targeted therapy.

**Table 1:** Targeted etiological therapies for vascular anomalies of the head and neck.

Drug	Primary pathway target	Key actionable genotypes	Best-fit head and neck phenotypes	Expected clinical effects	Major toxicities	Monitoring priorities
Sirolimus	mTOR	*PIK3CA*, *TEK* (TIE2), lymphatic pathway activation	Lymphatic malformations; venolymphatic malformations; complex lymphatic anomalies	Symptom stabilization; reduction in swelling, bleeding/oozing; selective volume reduction (strongest in pure lymphatic malformations)	Cytopenias; mucositis; hyperlipidemia; infection risk	CBC; CMP; lipid panel; sirolimus trough levels
Alpelisib	PI3Kα	*PIK3CA*	Severe PIK3CA-driven cervicofacial lymphatic and venolymphatic malformations; PROS-associated overgrowth	Lesion regression; reduction in overgrowth; improved function; decreased procedure burden	Hyperglycemia; rash; diarrhea; mucositis	Fasting glucose; HbA1c; electrolytes; weight; dermatologic exam
Trametinib	MEK1/2	*KRAS*, *NRAS*, *ARAF*, *MAP2K1*	Extracranial AVMs; central conducting lymphatic anomaly; RASopathy-associated lymphatic disease	Functional improvement; reduced lymphatic leakage; disease stabilization; selective lesion regression	Cardiomyopathy; ocular toxicity; rash; diarrhea; fatigue	Echocardiogram; ophthalmologic exam; dermatologic & gastrointestinal (GI) surveillance

## 2 Molecular architecture underlying actionable vascular malformations

**Figure 1:**
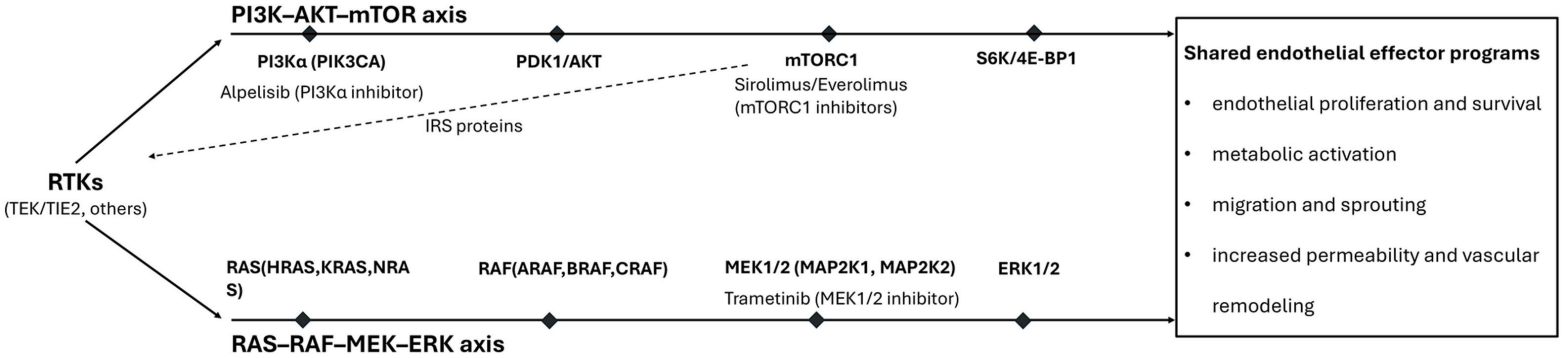
Actionable endothelial signaling architecture in vascular malformations and targets of etiological therapy. Most actionable vascular malformations converge on two endothelial signaling axes, PI3K-AKT–mTOR and RAS–RAF–MEK–ERK, which can be engaged by upstream receptor tyrosine kinases (RTKs) including *TEK*/*TIE2*. TEK/TIE2 and *PIK3CA* activating variants show strong coupling to PI3K pathway signaling in venous, lymphatic, and combined low-flow malformations, supporting pathway-matched therapy with mTOR inhibitors (sirolimus, everolimus) or PI3Kα inhibition (alpelisib). Activating variants in *HRAS*/*KRAS*/*NRAS*, *ARAF*/*BRAF*, and *MAP2K1/2* drive RAS/MAPK signaling in extracranial and brain arteriovenous malformations and complex lymphatic anomalies, supporting MEK inhibition (e.g., trametinib). Both pathways converge on shared endothelial effector programs, including proliferation/survival, migration/sprouting, permeability, and vascular remodeling, that underpin clinically significant disease, including lesions of the head and neck.

Building on this shift from descriptive phenotype to pathway-defined disease introduced above, current approaches emphasize underlying signaling pathways as a practical basis for therapeutic decision-making (1, 7). The most consistently actionable biology in vascular malformations converges on two dominant signaling axes, PI3K pathway and RAS/MAPK pathway (Figure 1) (7).

### 2.1 PI3K pathway signaling in vascular malformations

The PI3K pathway regulates endothelial growth, survival, metabolism, and permeability and is one of the most thoroughly characterized drivers of vascular malformation biology (7, 8). Class I PI3Kα, encoded by *PIK3CA*, generates phosphatidylinositol-3,4,5-trisphosphate (PIP₃) at the inner plasma membrane, recruiting AKT and PDK1 and leading to phosphorylation of AKT and activation of downstream targets including mTOR complex 1 (mTORC1) (9). In venous and lymphatic endothelium, this signaling axis promotes cell proliferation, survival, glycolytic metabolism, and increased vascular permeability, and perturbs normal vessel maturation and lumen architecture (8, 10).

Its involvement in human disease is most prominently established through activating somatic variants in *PIK3CA* and activating mutations in *TEK* (TIE2), a receptor tyrosine kinase that signals upstream into PI3Kα. Gain-of-function *TEK* mutations lead to ligand-independent TIE2 autophosphorylation, recruitment of the p85-p110α PI3K complex, and chronic AKT and mTORC1 activation in venous endothelial cells, producing ectatic, thin-walled venous channels that mirror sporadic venous malformation histology (8, 11). Somatic *TEK* mutations constitute a major cause of sporadic venous malformations, while activating *PIK3CA* variants account for a substantial fraction of venous malformations, particularly those lacking *TEK* mutations, and are central across a broader *PIK3CA*-related disease spectrum that includes lymphatic malformations, combined slow-flow malformations, and segmental overgrowth syndromes (8, 11, 12). Across this disease spectrum, accumulating data suggest that not all *PIK3CA* variants are equivalent. Recurrent hotspot substitutions in the helical and kinase domains (such as E542K, E545K, and H1047R) are repeatedly observed across venous malformations and *PIK3CA*-related overgrowth phenotypes, and genotype–phenotype differences have been reported across molecular subtypes of venous malformation (for example, differences in lesion localization and histologic features between *PIK3CA*-driven and TEK-driven disease) (8, 12–14). Emerging data in vascular anomalies and PROS cohorts further suggest the possibility of variant-level differences in clinical presentation and tissue involvement, although definitive correlations remain limited. This mirrors broader oncologic experience in which different *PIK3CA* mutations can have distinct biological effects and therapeutic implications within the PI3K pathway (9). Although vascular-anomaly-specific genotype–phenotype datasets remain relatively small, recognizing this potential heterogeneity is important, as future trials may incorporate variant-level stratification when evaluating PI3K- and mTOR-directed therapies. In experimental models, endothelial expression of hotspot *PIK3CA* variants drives abnormal venous and lymphatic vessel morphogenesis, tissue overgrowth, and leakage; pharmacologic inhibition of PI3Kα or mTORC1 reverses or attenuates these phenotypes, providing direct functional support for pathway causality (6, 8, 10).

This topology has direct therapeutic implications. mTOR inhibitors such as sirolimus and everolimus act downstream of PI3Kα by forming a complex with FKBP12 and allosterically inhibiting mTORC1, thereby suppressing S6 kinase and 4E-BP1 phosphorylation, reducing protein synthesis, cell growth, and aspects of permeability and inflammatory activation (15, 16). Clinically, this translates into symptom stabilization, reduced swelling and leakage, and partial volume reduction in many PI3K-AKT–mTOR-driven malformations, even when the upstream driver is *PIK3CA* or *TEK* (5, 17–19). However, because mTORC1 lies distal to PI3Kα and AKT, upstream signaling remains intact, and feedback circuits (for example, through IRS proteins) may limit the depth of pathway suppression (20, 21). In contrast, PI3Kα inhibitors such as alpelisib directly target the proximal catalytic subunit encoded by *PIK3CA*, more completely suppressing PIP₃ generation and AKT activation in *PIK3CA*-mutant cells. Translational and clinical data in *PIK3CA*-related overgrowth and lymphatic malformations support the concept that direct PI3Kα inhibition can produce more pronounced lesion regression and overgrowth control in *PIK3CA*-driven disease, particularly when mTOR inhibition yields only partial response (6, 9, 22). These mechanistic relationships explain why PI3K-AKT–mTOR biology is central to etiological therapy selection and why sequencing between mTOR and PI3Kα inhibition has become a key practical question in *PIK3CA*-driven head and neck malformations.

### 2.2 RAS/MAPK pathway signaling in vascular malformations

The RAS–RAF–MEK–ERK cascade is a core endothelial signaling axis that integrates inputs from multiple receptor tyrosine kinases and controls proliferation, migration, sprouting behavior, and vascular remodeling (23, 24). Activated RAS (*HRAS*, *KRAS*, *NRAS*) cycles to a GTP-bound state at the plasma membrane, recruits RAF kinases (*ARAF*, *BRAF*, *CRAF*), and initiates a phosphorylation cascade through MEK1/2 to ERK1/2 (23). In endothelial cells, sustained ERK activation alters arterial–venous identity programs, increases sprouting and shunting, disrupts normal mural cell investment, and can drive high-flow arteriovenous connections and pathological lymphatic remodeling (25–27). Although *TEK*/TIE2 can activate MAPK signaling in principle, pathogenic *TEK* variants preferentially recruit PI3K over GRB2-SOS complexes, resulting in only weak and transient ERK activation that is insufficient to drive RAS/MAPK-type malformation phenotypes.

Recurrent activating somatic variants in *KRAS*, *NRAS*, *BRAF*, and *MAP2K1* have been identified across multiple vascular malformation phenotypes (4, 28). Functionally, these mutations typically enhance GTP loading of RAS or increase MEK or RAF kinase activity, producing persistent ERK phosphorylation in lesional endothelium (28). Two high-impact examples illustrate the clinical relevance of this pathway with direct implications for head and neck disease. First, somatic activating *MAP2K1* mutations are a common cause of extracranial arteriovenous malformations, many of which involve the face, scalp, and cervical region (29). *In vitro* and *in vivo* models confirm that these *MAP2K1* variants increase MEK1 activity, augment ERK signaling, and drive the formation and expansion of arteriovenous shunts and nidus vessels, reproducing key features of AVM biology (29). Second, somatic activating *KRAS* mutations have been identified in a large proportion of brain arteriovenous malformations, where endothelial *KRAS* activation promotes ERK signaling, perturbs arterial–venous specification, and induces arteriovenous shunting in animal models, establishing a *KRAS*→ERK-driven endothelial mechanism for cerebrovascular malformations (25). Related work in complex lymphatic anomalies has extended this paradigm to lymphatic endothelium, with gain-of-function variants in *ARAF* and *NRAS* underlying central conducting lymphatic anomaly and kaposiform lymphangiomatosis, respectively, and demonstrating ERK-dependent lymphatic dysfunction that is reversible with MEK inhibition (26, 30).

These mechanistic insights are directly relevant to therapy because MEK1/2 represent a critical signaling checkpoint whose inhibition can uniformly dampen ERK pathway output, irrespective of the specific upstream RAS or RAF alteration. Trametinib, an allosteric MEK1/2 inhibitor, suppresses ERK phosphorylation downstream of diverse RAS, RAF, and MEK mutations without the need to target the specific upstream GTPase or kinase (31). In RAS/MAPK-driven lymphatic disease, MEK inhibition has been associated with resolution of lymphatic leakage, improvement in protein-losing enteropathy and effusions, and radiographic disease regression, consistent with normalization of ERK-dependent lymphatic endothelial behavior (26, 30, 32, 33). In AVM models and emerging clinical experience, MEK inhibition attenuates ERK signaling in lesional endothelium, reduces abnormal flow and shunt complexity, and stabilizes or partially regresses high-flow lesions that are otherwise refractory to procedural therapy (27, 29, 34). These data support a pathway-matched rationale in which identification of a RAS/MAPK driver, *KRAS*, *NRAS*, *ARAF*, *MAP2K1* or related variants, justifies consideration of MEK inhibition as an etiological strategy in selected, clinically significant head and neck malformations. At present, direct allele-specific RAS GTPase inhibitors used in oncology, such as *KRAS* G12C-targeted agents, have not yet been systematically evaluated in vascular anomalies, and the mosaic, non-G12C-dominant RAS mutational spectrum in these disorders means that currently available G12C-specific drugs would be applicable to only a very small subset of patients.

### 2.3 Somatic mosaicism, molecular diagnosis, and pathway assignment

For most vascular malformations, the causal variant is post-zygotic and therefore present in only a subset of cells, often enriched within lesional endothelium. This phenomenon of somatic mosaicism explains why lesions are focal or segmental, why blood testing is frequently negative, and why variant allele fractions (VAFs) may be very low even in affected tissue (28). Mosaicism is a major cause of missed molecular diagnoses in routine clinical workflows and a central barrier that modern precision frameworks must overcome.

These biological realities carry important implications for molecular diagnosis. While lesional tissue remains the highest-yield substrate for many malformations, because it is most likely to contain the mutant endothelial population at detectable levels, tissue sampling is often challenging in head and neck disease due to airway risk, hemorrhage risk, cranial nerve proximity, and aesthetic considerations. In this context, cell-free DNA (cfDNA) strategies have become increasingly valuable. cfDNA isolated from plasma or lesion-associated fluid (e.g., cyst fluid or lymphatic fluid) can detect low-level somatic variants and provide a minimally invasive route to molecular diagnosis, and in integrated workflows can complement or occasionally substitute for tissue sequencing when tissue is inaccessible or prior testing has been negative due to low VAF (35). In our integrated genomic profiling cohort (4), cfDNA from lymphatic fluid or plasma contributed to the molecular diagnosis in a subset of individuals with both primary complex lymphatic anomalies and other vascular malformations, with pathogenic variants detected in 19 of 47 cfDNA samples analyzed using ultra-deep targeted sequencing. These positive samples included patients with primary complex lymphatic anomalies (such as central conducting lymphatic anomaly and generalized lymphatic anomaly) and patients with slow-flow venous and combined malformations, often in the setting of substantial lesional burden or diffuse lymphatic involvement. By contrast, cfDNA was frequently negative in patients with very focal, well-circumscribed malformations and in acquired lymphatic conduction disorders associated with congenital heart disease, despite clear clinical and imaging evidence of disease. These patterns suggest that cfDNA yield is influenced by both lesional biology and overall disease burden, as well as by sampling site. In practice, yield correlates with overall disease burden (diffuse/multifocal lesions > focal lesions), and we have not observed reliable detection from plasma cfDNA in small, well-circumscribed superficial malformations in the absence of drainable lesion fluid. This study explicitly highlights limited tissue access, mosaicism, and sequencing depth as major obstacles to targeted therapy and demonstrates how deep genomic profiling and flexible sampling strategies can improve diagnostic yield and inform treatment selection (4).

Low VAF is the rule rather than the exception in many lesions. Studies of venous malformation genetics, for example, routinely report *PIK3CA* variants at low allele fractions in tissue, consistent with endothelial-restricted mosaicism (8). These observations have direct clinical consequences. Assay sensitivity becomes critical, as standard-depth next-generation sequencing may fail to detect variants in the 1–5% range, necessitating ultradeep sequencing or digital PCR for hotspot mutations. Specimen selection is equally important, as endothelial-enriched samples increase yield whereas mixed tissue dilutes the molecular signal. Pathway assignment can remain clinically actionable even when the exact molecular label is uncertain: once a lesion is confidently mapped to PI3K pathway or RAS-MAPK activation, etiological therapy selection becomes mechanistically rational.

Although ISSVA classification remains essential for diagnosis, communication, and procedural planning (1), it is not designed to function as a therapeutic algorithm. Multiple molecular etiologies can underlie a single phenotype, such as venous malformations driven by either *TEK* or *PIK3CA*, and conversely, shared pathway activation can unify diverse phenotypes under a common therapeutic logic, as seen across RAS/MAPK-driven malformation subtypes (7, 28). This pathway-based view provides the conceptual bridge from molecular diagnosis to the targeted therapies discussed in subsequent sections.

## 3 Targeting the PI3K-AKT–mTOR Axis

### 3.1 mTOR inhibitors

In vascular and lymphatic malformations, recurrent somatic *PIK3CA* variants and, in a subset of venous malformations, activating *TEK*/TIE2 mutations lead to hyperactivated PI3K pathway signaling, driving sustained endothelial growth, metabolic activation, increased permeability, inflammatory remodeling, and disordered vessel maturation (7). In this biological context, mTOR inhibition is employed as an etiological, disease-modifying strategy, intended not merely to palliate symptoms but to suppress the dysregulated signaling state that sustains lesion activity. mTOR inhibitors are particularly compelling in lymphatic disease biology because permeability abnormalities, inflammatory flares, progressive edema, and functional compromise, the hallmarks of many lymphatic malformations, are consistent with downstream effects of PI3K pathway hyperactivation. This is especially relevant in head and neck practice, where lymphatic malformations are most commonly diagnosed and frequently involve the airway, swallowing, and speech (36).

Among available agents, sirolimus (rapamycin) is the most widely used mTOR inhibitor in vascular malformation care and is supported by the most substantive clinical evidence base (5). Everolimus, a pharmacologically related compound, is sometimes employed when sirolimus is not tolerated or feasible; however, the vascular malformation evidence base for everolimus remains comparatively limited and is currently supported primarily by case-level experience and emerging clinical trials (37, 38).

Across published experience, the head and neck phenotypes most responsive to mTOR inhibition fall into three overlapping clinical categories. First, lymphatic malformations, including macro-, micro-, and mixed-cystic disease and infiltrative airway-adjacent lesions. In the PERFORMUS randomized observational-phase trial of children with slow-flow malformations, the subgroup with pure lymphatic malformations demonstrated the clearest signal toward lesion volume reduction, accompanied by improvements in bleeding, oozing, and quality of life (17). Second, venolymphatic and combined slow-flow malformations, which frequently show meaningful improvement in pain, oozing, bleeding, and quality of life even when volumetric MRI changes are modest (17).

Third, complex lymphatic malformations, which often present to head and neck teams through cervicothoracic involvement, edema, effusions, and compressive or functional sequelae, commonly benefit from mTOR inhibition as part of a multidisciplinary disease-stabilization strategy (5).

Prospective clinical evidence firmly establishes sirolimus as an effective systemic therapy for complicated vascular malformations. The pivotal Phase II trial by Adams and colleagues demonstrated high rates of clinical benefit, predominantly partial responses by composite outcome measures, accompanied by an acceptable safety profile (5). More recent controlled data refine expectations: in the PERFORMUS trial, sirolimus did not produce uniform volumetric reduction across all slow-flow subtypes, but did yield clinically meaningful improvements in symptoms, particularly pain, bleeding, and quality of life, with the most consistent volumetric signal observed in pure lymphatic malformations (17).

Genomic stratification further sharpens therapeutic expectations and escalation pathways. Integrated profiling studies demonstrate that actionable PI3K pathway and RAS/MAPK drivers occur across vascular malformation phenotypes and that identifying the dominant driver can rationalize both initial therapy and switching decisions.

From a practical standpoint, therapeutic drug monitoring is commonly employed. In the Adams Phase II trial, sirolimus was initiated at 0.8 mg/m^2^ twice daily with target trough concentrations of 10–15 ng/mL (5). Subsequent trials such as PERFORMUS adopted lower target ranges (4–12 ng/mL), reflecting evolving efforts to balance efficacy and toxicity (17). Longitudinal monitoring includes assessment for cytopenias, hepatic and renal dysfunction, metabolic abnormalities, particularly dyslipidemia, and mucosal toxicity (39). Inadequate response should be defined against patient-specific clinical threats and goals. Escalation is considered when progressive disease persists despite adequate therapeutic exposure, when functional endpoints fail to improve even if imaging is stable, when toxicity prevents maintaining therapeutic dosing, or when molecular results indicate an alternative dominant pathway, consistent with the genomics-guided framework articulated by Li et al. (4).

### 3.2 Direct PI3K inhibition for *PIK3CA*-driven disease

Direct PI3Kα inhibition is mechanistically most compelling when *PIK3CA* functions as the primary causal driver of the disease signaling hierarchy. For many slow-flow vascular malformations and overgrowth-associated vascular phenotypes, activating *PIK3CA* variants occupy this initiating position, and PI3Kα inhibition therefore targets the upstream oncogenic-like signal that propagates through AKT and mTOR to produce abnormal endothelial growth, vessel dilation, lymphatic dysfunction, and tissue overgrowth. This mechanistic proximity is the central reason PI3K inhibition is often considered more etiological than downstream suppression with mTOR inhibitors in *PIK3CA*-driven disease (6). Preclinical and translational studies reinforce this logic. In a *PIK3CA*-driven lymphatic malformation model and in human patients with severe *PIK3CA*-related lymphatic malformations, alpelisib produced marked lesion reduction and clinical improvement, including in individuals previously refractory to rapamycin-based strategies (6).

Alpelisib is currently the principal PI3Kα inhibitor used for *PIK3CA*-driven vascular malformations. Alpelisib (BYL719) is an oral, selective PI3Kα inhibitor originally developed for oncology; it received FDA approval in 2019 for *PIK3CA*-mutated breast cancer and, on April 5, 2022, accelerated approval for adult and pediatric patients ≥2 years with severe manifestations of *PIK3CA*-related overgrowth spectrum (PROS) requiring systemic therapy (40, 55). This regulatory milestone is highly relevant to head and neck vascular malformation care because many clinically severe cervicofacial phenotypes, particularly combined vascular malformations with overgrowth, fall within PROS and are enriched for *PIK3CA* drivers (41). In the head and neck region, *PIK3CA*-driven disease often presents as a continuum of phenotypes rather than a single lesion category. Common clinical presentations include: (1) Cervicofacial lymphatic malformations (macro-, micro-, and mixed cystic), frequently with airway or swallowing compromise (41); (2) Combined venolymphatic malformations with infiltrative disease, recurrent swelling, pain, and bleeding or oozing, sometimes with progressive overgrowth (42); (3) Head and neck PROS phenotypes, including infiltrating lipomatosis and locoregional overgrowth with associated vascular malformations, where lesion biology is *PIK3CA*-driven and therefore directly targetable (41). In practice, the indication for systemic etiological therapy in head and neck disease is often determined less by lesion label and more by severity, functional threat, recurrence after procedures, and evidence of pathway-driven progression, features that are common in *PIK3CA*-driven cervicofacial disease.

Clinical evidence supporting alpelisib now spans translational models, head-and-neck-focused cohorts, and large real-world datasets. Delestre et al. (6) provided translational proof-of-concept and early clinical validation. Wenger et al. (41) reported that individuals with head and neck PROS treated with alpelisib experienced reduced malformation size and locoregional overgrowth, improved symptoms and function, and decreased need for invasive procedures. The EPIK-P1 real-world study further confirmed clinical benefit and durability of response across PROS manifestations (14). Across studies, responses include reductions in swelling, pain, bleeding/oozing, functional compromise, and frequently measurable decreases in lesion burden, although kinetics and volumetric regression vary by lesion composition and baseline severity (42, 43).

Toxicity and sequencing considerations for alpelisib are particularly important in vascular malformation care because treatment is often prolonged and initiated in childhood. Because PI3Kα signaling is central to insulin signaling, hyperglycemia is the most common and clinically significant adverse effect. Rash and diarrhea are also frequent and may be dose-limiting. These toxicities are well documented in FDA labeling (22) and a clinical review by the Canadian Agency for Drugs and Technologies in Health (CADTH; now Canada’s Drug Agency) (44). For vascular malformation patients, often pediatric and on long-term therapy, practical management requires baseline metabolic assessment, early intensive glucose monitoring, proactive dermatologic surveillance, and longitudinal metabolic follow-up, frequently involving coordinated care among vascular malformations, endocrinology, and dermatology teams. Two sequencing approaches dominate contemporary practice. mTOR inhibitor first, then escalate to PI3K inhibition is common when molecular data are incomplete, tissue is inaccessible, or initial goals emphasize symptom stabilization. Escalation to alpelisib is considered once *PIK3CA* is confirmed or when mTOR inhibition proves inadequate or intolerable, consistent with translational evidence demonstrating alpelisib efficacy in rapamycin-refractory patients (6). Upfront PI3K inhibition is increasingly appropriate for confirmed, severe *PIK3CA*-driven head and neck disease, particularly when airway, swallowing, vision, disfigurement, or rapid progression dominate the clinical picture and procedural options are limited or high-risk (41). In both strategies, therapy selection is increasingly governed by driver confirmation and pathway assignment, reflecting the genomics-to-treatment framework demonstrated by Li et al. (4).

## 4 Targeting the RAS/MAPK pathway: MEK inhibitors

A major conceptual advance of the past decade has been the recognition that many vascular malformations, particularly high-flow arteriovenous malformations (AVMs) and subsets of complex lymphatic disorders, are driven by somatic activating variants that converge on ERK activation, reframing these conditions as signaling-driven disease and providing a direct rationale for MEK-targeted therapy (29). This insight is especially relevant to head and neck disease, where AVMs of the face, scalp, and oral cavity can be locally destructive, prone to recurrence after procedural therapy, and extremely difficult to eradicate surgically without significant morbidity. Somatic *MAP2K1* mutations are common in extracranial AVMs and increase MEK1 activity, providing a direct mechanistic rationale for inhibiting MEK as etiological therapy (29). Similarly, somatic activating *KRAS* mutations have been identified in the majority of analyzed brain AVMs, establishing a *KRAS*→ERK disease mechanism in cerebrovascular malformations (25).

On the lymphatic side, RAS/MAPK activation has been linked to severe phenotypes across the complex lymphatic malformation spectrum. A striking example is the identification of a recurrent gain-of-function *ARAF* variant in central conducting lymphatic malformation, where pathway-matched MEK inhibition produced dramatic clinical and radiographic improvement (26). Additional reports demonstrate successful trametinib therapy in *NRAS*-driven kaposiform lymphangiomatosis, including cases where the causative mutation was detected via plasma cell-free DNA, underscoring the importance of molecular diagnostics in difficult-to-biopsy disease (30).

Trametinib, an oral MEK1/2 inhibitor with extensive oncology experience and a well-characterized safety profile, has emerged as the dominant MEK inhibitor in the vascular malformation literature. Its utility now spans lymphatic disorders, AVM biology, and RASopathy-associated lymphatic disease, where clinically meaningful benefit has been repeatedly documented when lesions are driven by RAS/MAPK activation (26, 32, 33). Genomics-directed MEK inhibition follows a consistent therapeutic logic: benefit is strongest when genetic or pathway evidence of MAPK activation is present, such as *KRAS*, *NRAS*, *ARAF*, or *MAP2K1* variants. This paradigm is explicitly articulated in large-scale integrated profiling studies, which highlight mosaicism, tissue inaccessibility, and insufficient sequencing depth as major barriers and demonstrate how systematic genomic profiling can directly inform both diagnosis and targeted treatment selection across vascular malformation phenotypes (4).

Clinically, genomics-directed MEK inhibition has been associated with resolution of lymphatic leakage and improved function in severe lymphatic disease (26), correction of coagulopathy and pulmonary compromise in *NRAS*-driven kaposiform lymphangiomatosis (30), and mechanistic support for MEK targeting in AVM subsets harboring *MAP2K1* or *KRAS* drivers (25, 29). Preclinical modeling further strengthens biological plausibility: in a *KRAS*-driven mouse model of Gorham-Stout disease, trametinib suppressed pathological lymphatic remodeling and reversed disease manifestations, reinforcing the concept that MEK inhibition can be disease-modifying in RAS/MAPK-driven lymphatic pathology (27).

Because vascular malformation patients, often pediatric, may require prolonged therapy, long-term safety strategy is essential. The trametinib prescribing information highlights toxicity domains particularly relevant to chronic non-oncologic use, including cardiomyopathy with decreased left ventricular ejection fraction, retinal disorders, dermatologic toxicity, gastrointestinal adverse effects, and rare but serious interstitial lung disease (31). In practice, long-term management typically includes baseline and periodic echocardiography, baseline ophthalmologic evaluation with prompt assessment of new visual symptoms, proactive dermatologic and gastrointestinal management to maintain adherence, and explicit plans for maintenance, tapering, and relapse management, recognizing that many lesions recur upon discontinuation and may require re-initiation of therapy.

## 5 Implementing precision etiological therapy in clinical practice

### 5.1 Pathway-guided workflow in head and neck disease

A practical precision-medicine workflow for vascular malformations begins with clinical risk triage, particularly in head and neck disease where airway compromise, vision-threatening progression, hemorrhage, and neurologic compromise may mandate urgent stabilization, while proceeding in parallel along two complementary diagnostic tracks. The first track is phenotype/anatomic definition, anchored in ISSVA classification and high-quality imaging (ISSVA taxonomy + MRI/MRA/ultrasound). The second track is molecular driver assignment, using genomics to map the lesion to a dominant signaling axis. In contemporary multidisciplinary centers, therapeutic selection is increasingly anchored to pathway identity rather than to descriptive lesion labels alone, consistent with the clinical reality that actionable drivers span phenotypes and that symptom control may be achievable even when volumetric regression is modest (17).

In practice, a commonly used pathway-driven algorithm follows several reproducible steps. First, clinicians confirm lesion class and urgency (slow-flow vs. high-flow; tumor vs. malformation; anatomic extent and functional threat) using clinical course and imaging, often relying on MRI-based characterization for slow-flow disease and MRA/angiographic strategies for high-flow lesions. Second, the team obtains molecular testing using the best-available substrate and assigns a dominant signaling axis: lesions mapping to the PI3K pathway (e.g., *PIK3CA* or *TEK*/TIE2 drivers) support selection of an mTOR inhibitor or, in *PIK3CA*-driven disease, PI3Kα inhibition; lesions mapping to the RAS/MAPK pathway (e.g., *KRAS*/*NRAS*, *MAP2K1*, BRAF, *ARAF*) support MEK inhibition. Large-scale integrated profiling work demonstrates the feasibility of this approach across diverse phenotypes, explicitly linking deep genomic profiling and flexible sampling to changes in diagnosis and targeted treatment selection (4). Third, the team sets explicit goals and defines thresholds for success or failure up front, emphasizing functional endpoints first in head and neck disease (airway, swallowing, speech, vision, bleeding/oozing, pain, infection frequency, quality of life), reflecting controlled-trial experience that symptom control may be the dominant benefit even without large MRI volume reduction (5, 17). Finally, patients are reassessed at predetermined intervals and therapy is escalated, switched, or de-escalated based on response, toxicity, and updated molecular evidence, keeping the pathway-to-drug logic explicit in decision-making (Table 2) (4).

**Table 2:** Practical triggers for therapeutic escalation and switching.

Clinical/molecular scenario	Action	Rationale
Partial clinical response on sirolimus with persistent functional compromise	Reassess molecular driver; consider escalation to PI3K inhibition	mTOR inhibition may provide symptom control but insufficient pathway suppression in PIK3CA-driven disease
Documented mTOR resistance and confirmed *PIK3CA* mutation	Switch to alpelisib	PI3Kα inhibition targets the proximal driver in PIK3CA-driven lesions
Identification of RAS/MAPK driver (*KRAS, NRAS, ARAF, MAP2K1*)	Initiate MEK inhibitor (e.g., trametinib)	Direct pathway-matched etiological therapy
Rapid progression, airway compromise, or major functional threat	Combine targeted medication with IR and/or surgery	Medical therapy stabilizes disease biology while procedural therapy controls immediate risk

### 5.2 Genomic sampling, mosaicism, and assay strategy

Because actionable variants in vascular malformations are frequently somatic mosaic, integrating genomics into routine care requires treating sampling strategy and assay sensitivity as core clinical decisions rather than secondary technical details. Integrated profiling studies underscore that limited tissue access, mosaicism, and inadequate depth are recurring barriers to actionable diagnosis and pathway-matched therapy (4). Practically, the highest-yield specimen should be pursued when safely obtainable, recognizing that lesional tissue often provides the best sensitivity for low-level mosaic variants. When biopsy risk is high, as is common in head and neck lesions, we would not advocate stand-alone biopsy performed solely for genomic characterization if it would require general anesthesia in an otherwise clinically stable child. This is particularly true for cervicofacial lymphatic malformations that are not scheduled for resection or other intervention. We instead prioritize opportunistic sampling at the time of clinically indicated procedures (such as debulking, tracheostomy revision, or sclerotherapy) and apply the tiered sequencing strategy developed in our genomic profiling program: when lesional tissue cannot be obtained safely, we rely on unconventional samples, including lymphatic fluid-derived CD31^+^ endothelial cells and cfDNA from lymphatic fluid or plasma. In our cohort of 356 individuals with vascular anomalies, this tiered approach, incorporating liquid-biopsy-based sampling, yielded a molecular diagnosis in 41% of participants with primary complex lymphatic anomalies and 72% of those with other vascular malformations, and informed medical therapy decisions in diagnosed patients, including initiation or modification of targeted treatment (4). Accordingly, we reserve anesthesia-requiring diagnostic biopsies for circumstances in which a molecular result is expected to alter management in the near term, such as clarifying whether PI3K versus RAS/MAPK pathway activation predominates when this distinction would affect systemic therapy or clinical trial eligibility. This risk–benefit framework is particularly salient in head and neck disease, where airway vulnerability and cumulative anesthetic exposure must be balanced against the incremental value of additional tissue.

cfDNA-based strategies provide a particularly valuable, minimally invasive route to molecular diagnosis. In practice, cfDNA currently complements rather than replaces tissue-based testing: a positive result may obviate the need for high-risk biopsy in patients with genomically informative lesions, but a negative cfDNA result does not exclude an underlying mosaic driver and should be interpreted in the context of lesion burden, sampling site (plasma versus lesion fluid), and overall pre-test probability (4, 35). Zenner et al. demonstrate the utility of cfDNA as a diagnostic analyte in vascular malformations, expanding access to molecular diagnosis when tissue is inaccessible or prior testing is negative because of low variant allele fraction (35). In either substrate, variant allele fraction should be interpreted in context: low VAF does not imply low biological relevance and often reflects endothelial-restricted mosaicism; deep sequencing and orthogonal confirmation (e.g., ddPCR) can be decisive when clinical suspicion is high (4). For complex lymphatic malformations, which frequently intersect cervicothoracic disease and may present to head and neck teams, consensus guidelines emphasize structured multidisciplinary evaluation and targeted diagnostic testing, providing a practical backbone for integrating genomics into a comprehensive care pathway (45).

### 5.3 Response assessment, procedural integration, and maintenance

Implementation of precision therapy often fails when response assessment is vague. A robust approach triangulates clinical, radiographic, and laboratory endpoints. Clinically, head and neck care prioritizes airway symptoms (including need for airway support), dysphagia/feeding status, speech impact, pain and pain crises, bleeding/oozing, cellulitis/infection frequency, and patient-reported quality of life, with endpoint selection informed by trial templates that foreground pain, bleeding/oozing, and quality of life and clarify that volumetric MRI response may be subtype-dependent (17). Radiographically, standardized MRI protocols and, when feasible, volumetric analysis support longitudinal comparison in slow-flow disease, while high-flow lesions may require flow-related endpoints (e.g., shunting burden, nidus devascularization), interpreted alongside functional outcomes and procedural morbidity. Laboratory endpoints are phenotype-dependent and may include markers of localized intravascular coagulopathy in venous/venolymphatic disease and albumin/immunoglobulin trends when protein-losing enteropathy or severe lymphatic leakage is present, particularly in complex lymphatic disorders (45).

Vascular malformations of the head and neck require particularly careful implementation of etiological therapy because modest lesion progression can threaten critical structures and because procedural morbidity is amplified in this region. Airway involvement is a dominant concern: cervicofacial lymphatic and venous malformations may cause acute or progressive obstruction and complicate airway management, often necessitating urgent multidisciplinary coordination and stabilization strategies before definitive intervention (46, 47). Proximity to cranial nerves and sensory organs increases the stakes of lesion growth and intervention, elevating the risk of irreversible vision or hearing impairment and cranial neuropathies, and reinforcing the need for early stabilization when progression is evident (3). The neurovascular density of the region increases hemorrhage and nerve-injury risk during procedures, particularly for high-flow lesions, supporting an approach in which systemic therapy is used when needed to reduce activity, improve tissue planes, or decrease recurrence risk (48). Beyond physical morbidity, head and neck malformations often impose substantial aesthetic and psychosocial burden across childhood and adulthood, making symptom stabilization and reduction in procedure burden clinically meaningful outcomes in their own right (47, 48). Because complete surgical resection is frequently constrained by adjacent vital structures and reconstruction challenges, a common practical goal is to stabilize or shrink lesions before IR or surgery, thereby improving procedural safety and reducing the number of stages required (41).

For most head and neck vascular malformations, optimal outcomes usually arise from intentional integration of systemic etiological therapy with interventional radiology (IR) and/or surgery rather than viewing these modalities as mutually exclusive. Contemporary multidisciplinary reviews emphasize that planning must balance function, cosmesis, recurrence risk, and procedure-related morbidity (48, 49). Common combination patterns include neoadjuvant targeted therapy to reduce inflammation, leakage, and bulk prior to sclerotherapy or debulking in infiltrative cervicofacial disease, and adjunct targeted therapy after IR/surgery to suppress the underlying pathway-driven biology that fuels recurrence or progression. Head and neck PROS cohorts treated with alpelisib report reduced invasive procedure burden alongside symptomatic and volumetric improvement, supporting this integrated strategy (41). For high-flow AVMs, staged embolization remains foundational, with ethanol sclerotherapy and embolization approaches described in head and neck series, while pathway-directed therapy is increasingly discussed as a future adjunct in refractory, genomically defined subsets (50, 51).

Because many lesions are chronic and mosaic, durability and relapse management are central real-world issues. Longitudinal cohorts show that symptom recurrence after discontinuation of sirolimus is common in subsets of slow-flow malformations, with improvement after re-initiation in many patients, supporting a “maintenance mindset” for selected severe phenotypes (18, 19). Emerging prospective trial evidence also supports the clinical reality of relapse in some patients after stopping therapy and emphasizes the importance of planning taper/holiday criteria and re-treatment thresholds (52). A pragmatic sequencing approach therefore begins with the most appropriate pathway agent given driver and urgency (often sirolimus when the driver is unknown and lymphatic symptom control is the immediate goal), escalates or switches when response is inadequate or toxicity limits exposure, and explicitly defines maintenance strategies for responders at the lowest effective exposure, with clear criteria for taper and relapse-triggered re-initiation (4, 5).

### 5.4 Long-term safety and ethical considerations

Precision etiological therapy in vascular malformations must address long-term safety and ethical considerations, particularly because most agents were developed in oncology and many uses remain off-label. Implementation is best supported by standardized adverse-event surveillance and multidisciplinary toxicity management (e.g., endocrinology collaboration for PI3K inhibitor-associated hyperglycemia; cardiology and ophthalmology involvement for MEK inhibitor monitoring), with careful developmental and risk–benefit assessment given long treatment durations in non-malignant disease (34, 53). Informed consent should be explicit regarding evidence level, alternatives, and uncertainty in long-term pediatric outcomes, and participation in registries and trials should be prioritized when feasible to strengthen generalizable evidence and equitable access to molecular diagnostics and targeted therapeutics (34, 53).

## 6 Conclusions and future directions

Despite major progress, several barriers still limit the consistent delivery of molecularly guided etiological therapy, particularly in head and neck disease where tissue access, procedural risk, and functional stakes are high. A dominant challenge remains incomplete molecular diagnosis, especially in mosaic disease. Many actionable variants are somatic and present at low variant allele fraction (VAF), and detection frequently fails because affected tissue is inaccessible, sequencing depth is insufficient, or sampling does not adequately capture mutant endothelial populations. A second barrier lies in phenotype–genotype complexity that exceeds “one label → one drug.” ISSVA classification remains indispensable for standardized diagnosis and communication, yet it is intentionally designed as a living, evolving classification whose treatment implications increasingly depend on molecular drivers that cut across diagnostic categories (1, 2). This disconnect between descriptive taxonomy and mechanistic therapy continues to complicate clinical decision-making. A third unresolved problem is choosing the optimal initial agent and sequencing strategy even after pathway identification. Clinicians must still navigate mTOR versus PI3K inhibition in *PIK3CA*-driven disease, determine when MEK inhibition is indicated, integrate systemic therapy with IR or surgery, and define “inadequate response” when functional outcomes improve but lesion volume remains stable. A fourth persistent challenge is long-term safety and durability in children, as these therapies are often initiated early in life, continued for years, and used in non-malignant disease, with limited prospective data guiding optimal dosing, monitoring intervals, taper strategies, and late-effect surveillance.

Emerging technologies are positioned to move the field from “best-effort precision” to routine precision. cfDNA already demonstrates the ability to deliver molecular diagnoses without surgery, directly addressing one of the largest access barriers in head and neck disease (35). Future work should standardize specimen selection (plasma vs. lesional or cyst fluid), sequencing depth and VAF reporting, orthogonal confirmation, and how cfDNA results are operationalized in treatment algorithms and clinical trials. At the discovery level, single-cell and spatial profiling promise to explain biological heterogeneity and reveal new therapeutic targets beyond the core PI3K-mTOR and RAS-MAPK axes. Single-cell transcriptomic studies of *PIK3CA*-driven models have begun to identify disease-relevant endothelial subtypes and immune-endothelial interactions that could inform future interventions (10). Complementary single-cell methodologies in *PIK3CA*-altered cells further illustrate how cell-type-restricted mutant expression can be missed by bulk approaches, reinforcing the rationale for higher-resolution diagnostics and target discovery (13). When genetic data remain ambiguous, very low VAF, variants of uncertain significance, or mixed lesions, the field is increasingly turning toward functional testing using endothelial models, organoids, and animal systems to confirm pathway activation and predict drug responsiveness, particularly for rare RAS/MAPK drivers and combined phenotypes in which pathway dominance is not evident from imaging alone.

Moving from pioneering case success to predictable clinical benefit will require standardization in at least four domains. Diagnostic protocols must incorporate routine ISSVA terminology, structured head and neck imaging pathways, and center-level standard operating procedures for molecular sampling, sequencing depth, and reporting thresholds (1). Therapy-selection algorithms should formalize pathway-first decision trees that integrate disease severity, urgency, lesion accessibility, and molecular evidence strength, with harmonized criteria for escalation and switching. Response assessment must converge on consensus endpoint sets tailored to head and neck priorities, airway and swallowing function, vision, bleeding, pain, and patient-centered quality of life, supplemented by standardized volumetric MRI when feasible. Finally, care delivery models must preserve and strengthen interdisciplinary team structures, as coordinated pathways demonstrably improve diagnostic accuracy and treatment planning in head and neck vascular malformations (54).

In conclusion, the central message of this review is that etiological medications are most rational and most effective when treatment selection is anchored to pathway biology rather than descriptive phenotype alone. Large-scale integrated genomics has established the feasibility of mechanism-matched therapy (4), while parallel therapeutic evidence confirms that pathway inhibition can be disease-modifying in appropriately selected patients. For head and neck vascular malformations, where functional stakes are uniquely high and surgical tolerance is limited, the next phase of progress depends on making this precision framework standard rather than exceptional: scalable molecular diagnostics including cfDNA, evidence-based sequencing strategies, and shared response metrics that reflect patient-centered outcomes. Together, these advances are redefining vascular malformation care from lesion control toward true mechanism-based disease modification, with particularly transformative implications for patients with head and neck involvement.
